# Midwifery

**Published:** 1854-05

**Authors:** 


MIDWIFERY.—*An account of seventeen cases of Parturition in which Chloroform was inhaled with pernicious effects.*—Dr. ROBERT LEE read before the Royal Medical and Chirurgical Society an account of seventeen cases of parturition in which chloroform was inhaled with pernicious effects. In the cases related, the injurious effects of the inhalation of chloroform were as follows:—In seven cases, insanity and great cerebral disturbance followed its exhibition. The use of the forceps was rendered necessary in five cases. In two, the contractions of the uterus were arrested, and the operation of craniotomy was performed. Peritonitis or phlebitis ensued in four cases. Epilepsy or dangersus fits of syncope supervened in two instances. Many analogous cases had been confided to the author by friends; and public rumor swelled the size of the chapter of accidents; but he wished merely to give accounts of those which had come under his own observation. The author strongly animadverted upon the levity and thoughtlessness which had accompanied the use of this subtle and dangerous poison. Soon after its discovery, before the amount of its power, and even its composition, had been fully understood, he had been horrified by the announcement of its application to midwifery, and he then prophesied that deplorable results would ensue—a prognostication which experience had unhappily proved to be correct. It was natural that women, doomed to bring forth their young in pain and sorrow, should seek every means by which they might palliate the anguish they suffer; and instances related in which the process of parturition had been effected without pain, served to render nugatory the unwelcome admonitions of those who pointed to the evils that might occur. The author expressed his opinion, that the most serious effects which arose from the inhalation of this agent, were, first, languid and inefficient uterine contractions; secondly, a greater susceptibility to the risks that arose from inflammation and fever. In spite of the fact that grave and experienced physicians had expressed their concurrence with the author's views, yet the question, whether chloroform should be inhaled had become almost extra-professional; as silly and ignorant women of fashion chose to set the example of using it, the cause of science and humanity thus being in the hands of the most weak and frivolous portion of the community; and, as there was a systematic concealment of the truth by the physicians who used it, he feared that young and inexperienced mothers would still be lured to their destruction. In conclusion, the author expressed a hope that his essay might tend to rescue the profession from the dominion of an ignominious and disgraceful practice.

Dr. Snow regretted that Dr. Lee had indulged in such severe remarks as those which occupied the concluding portion of his paper, more particularly as he felt great admiration for the talent displayed in the essay, and great respect for the honest and manly spirit that caused the author to attack anything which he considsidered injurious either to a patient under treatment, or to the cause of science generally. Dr. Lee certainly had related the history of several distressing cases; but, in his (Dr. Snow's) opinion, not one of the symptoms described could be referable to the exhibition of chloroform. Dr. Lee must have known that chloroform was a most volatile spirit, and that half an hour after its application no traces of it could be found in the system; and yet, in some of the cases related it was stated, that inflammatory action had arisen two or three, and even as much as fourteen days after labor. How could the evil effects described be laid, with any show of probability, to the noxious influence of chloroform? Some of the symptoms mentioned in the cases might be naturally referred to hysteria. Dr. Lee had stated, that the chloroform checked the uterine contractions; this statement Dr. Snow could contradict, as, in the many cases in which he had superintended its employment, he had continually felt the uterus contracting firmly. The amount of the spirit inhaled in obstetric practice was trifling, compared with that taken during surgical operations. One great benefit derived from its application was, that it tended to do away with the use of laudanum; and Dr. Lee ought to hail this fact with joy, inasmuch as the time that the former drug remained in the system was so short in comparison with the latter. In conclusion, Dr. Snow expressed his opinion, that, notwithstanding the recorded cases of death having occurred from the use of chloroform, which now amounted altogether to about thirty-seven, it had been the means of saving many lives, by preventing nervous excitability consequent upon severe operations.

Dr. Gream, with great respect to the learning of the author of the paper, was of opinion that he had, in this, as in many other cases, preferred antiquated measures to modern discoveries, merely because the latter were modern; and had given a list of extreme cases. He entered into the question, as to whether uterine contraction was checked by the inhalation of chloroform; and stated, that when a large dose was given, it did so favorably when the patient was exhausted by a long and painful labor. He announced that he was only a recent convert to the practice of giving ether or chloroform in midwifery.

Dr. Merriman related a case where chloroform was administered during labor. Pain ceased, and he returned home for a short time. On being called again to see the patient, after a lapse of two hours, having been told that she was very ill, he was unable to find any trace of the child; and the systemic effect was so violent, that it was long before labor recommenced, the uterus having been temporarily paralyzed. She never recovered from the effects of the narcotic, and was finally removed to a lunatic asylum. This case had so alarmed him, that he had never since given his assent to the employment of chloroform.

Dr. Chowne said, the subject had been so talked about publicly, that it had become extra-professional. He expatiated lengthily upon the history of chloroform, remarking, that in his opinion, it had been received as a professional remedy without sufficient inquiry having been made into its properties. He instanced as an example, the first check given to its unconditional application, which was owing to a death which had occurred when the patient was undergoing the operation of having a tooth removed. Disease of the heart having been discovered in that case, a rule was laid down that chloroform should not be administered when an ailment of that organ was existing. Dr. Chowne gave some more instances in illustration of his opinion, and finally referred to Dr. Merriman's patient, whom he had seen; and stated, that the ergot of rye had been administered after the chloroform, without having any action on the uterus.

Mr. Carter said, that seventeen cases on which to found a decided opinion whether chloroform was beneficial or otherwise in midwifery, were not enough. He had frequently used it himself with no evil results, except in two cases, in one of which the patient was in a very asthenical condition. In the other case, death ensued while the woman was under operation. He reminded Dr. Lee that it was frequently used in country practice.

Mr. Ferguson stated that being engaged exclusively in surgical practice, he was unable to offer any opposition to Dr. Lee and his statements; yet he wished to know how it was, that these terrible results described, of insanity, phlebitis, and prolonged syncope, did not occur after the inhalation of chloroform during surgical operations. He considered that Dr. Chowne had not drawn a sufficient distinction between surgical and obstetrical practice, as regards the use of this narcotic. He could not understand how paralysis of the uterus could be caused by chloroform, when fæces and urine were often forcibly expelled when the patient was under its influence, thus showing that it does not destroy involuntary muscular action. He expressed his opinion, that our experience in England of the good or evil effects of chloroform was far too limited for a decided opinion to be given on the subject; and referred Dr. Lee to the extensive use that is made of it with beneficial results in the United States.

Dr. Lee, in reply, stated that there was a vast difference between the birth of a child and the extraction of a tooth, or a stone from the bladder, or any other surgical operation; the latter being dependent upon external manual action, the former being a work of nature, dependent upon nervous influence. The child is expelled from the uterus by regular contractions, and the exhibition of chloroform stops this action, as he had seen and as he had heard from competent persons, who had attended cases where it had been administered. Moreover, he wished to know what was the dose of the drug that should be used. It was a pretence, merely to sprinkle a few drops of the liquid upon a handkerchief, and hold it over a patient's nose—a process which he designated as "chloroform ã la Reine." And when the forceps were necessary, how could the medical attendant apply them with any safety when the woman was lying writhing about on the bed in convulsions following the use of this deleterious compound? No—woman was to bring forth her young in pain and with sorrow. This was a Divine ordinance, doubtless founded with a wise and merciful intention; and he considered that the continuance of the discreditable and cowardly practice of seeking relief from a necessary suffering, by resorting to such measures as he was combating, would be fraught with danger to her, not only physically, but also morally.

The Society adjourned at the usual hour.—*Medical Times and Gazette.*

